# Supplementary material for: The interactome of intact mitochondria by cross-linking mass spectrometry provides evidence for coexisting respiratory supercomplexes
Source: Mol Cell Proteomics. 2017 Dec 8;17(2):216–32. doi: 10.1074/mcp.RA117.000470 (PMC5795388; doi:10.1074/mcp.RA117.000470)
Supplement: Supplemental Data [file supp_RA117.000470_133922_0_supp_23978_fzffwf.zip › spectra_annotation/mito_spectra_annotation/105-1-19-1-12-1.pdf]

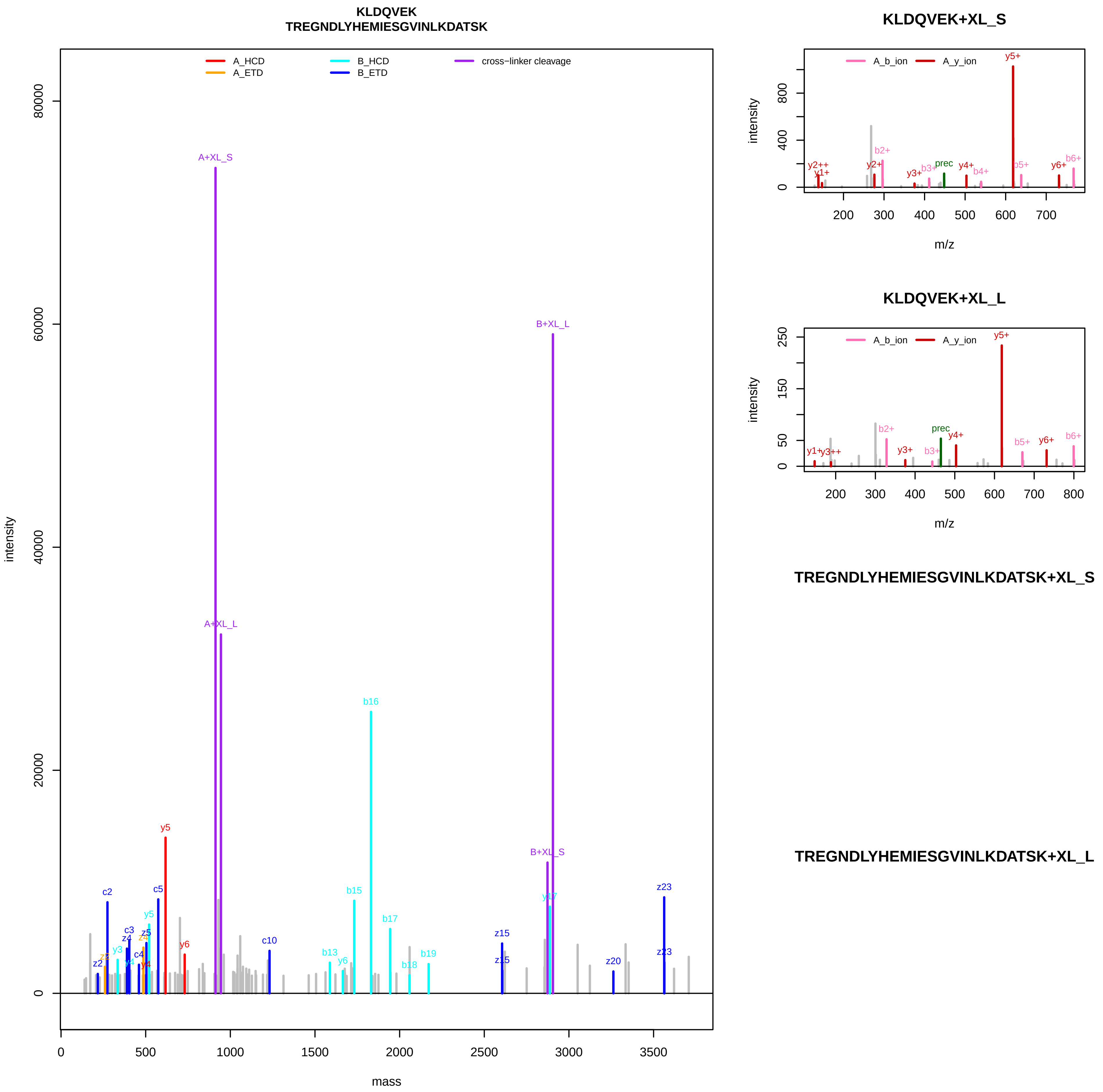
KLDQVEK
TREGNDLYHEMIESGVINLKDATSK
A_HCD
A_ETD
B_HCD
B_ETD
cross−linker cleavage
A+XL_S
A+XL_L
B+XL_L
B+XL_S
b13
b15
b16
b17
b18
b19
c2
c3
c4
c5
c10
y3
y4
y5
y6
y17
z2
z4
z5
z15
z20
z23
intensity
mass
0
20000
40000
60000
80000
500
1000
1500
2000
2500
3000
3500
KLDQVEK+XL_S
A_b_ion
A_y_ion
y2++
y1+
y2+
b2+
y3+
b3+
prec
y4+
b4+
y5+
b5+
y6+
b6+
intensity
m/z
0
400
800
200
300
400
500
600
700
KLDQVEK+XL_L
A_b_ion
A_y_ion
y1+
y3++
b2+
y3+
b3+
prec
y4+
y5+
b5+
y6+
b6+
intensity
m/z
0
50
150
250
200
300
400
500
600
700
800
TREGNDLYHEMIESGVINLKDATSK+XL_S
TREGNDLYHEMIESGVINLKDATSK+XL_L
